# Prevalence of hepatitis B and hepatitis C infection in Libya: results from a national population based survey

**DOI:** 10.1186/1471-2334-14-17

**Published:** 2014-01-09

**Authors:** Mohamed A Daw, Abdallah El-Bouzedi

**Affiliations:** 1 Department of Medical Microbiology, Faculty of Medicine, Tripoli, Libya; 2 Department of Laboratory Medicine, Faculty of Biotechnology, Tripoli, Libya; 3 Professor of Clinical Microbiology & Microbial Epidemiology, Acting Physician of Internal Medicine, Scientific Coordinator of Libyan National Surveillance Studies of Viral hepatitis & HIV, Tripoli, Libya

**Keywords:** Hepatitis B virus, Hepatitis C virus, Libya, Prevalence, HBsAg, Anti-HCV-Ab

## Abstract

**Background:**

Libya is one of the largest countries in Africa and has the longest coast in the Mediterranean basin facing southern Europe. High rates of prevalence of viral hepatitis have been observed in various regions in Africa, but the prevalence in Libya is not well documented. We report on a large-scale nationwide study that evaluated the epidemiology of hepatitis B and hepatitis C in Libya and assessed the risk factors involved.

**Methods:**

A cross-sectional study was carried out in 2008 on 65,761 individuals all over Libya. The country was divided into 12 regions according to the population density and sampling within each region was carried out under the supervision of the National Centre for Prevention of Infectious Diseases. Serum samples were collected from both males and females of all ages in both urban and rural areas and tested for HBsAg for hepatitis B and anti-HCV antibody for hepatitis C. Prevalence rates were determined in regions and in different groups and correlated with different demographic and risk factors involved in the spread of these viruses.

**Results:**

The prevalence of hepatitis B and hepatitis C viruses varied regionally across the country. The overall prevalence of hepatitis B was 2.2% (95% CI 2.1%-2.3%) and was higher among males than females (1.4:1.0). Hepatitis C virus (HCV) prevalence was 1.2% (95% CI 1.1-1.3) and it increased gradually after the age of 30 years (0.7-0.9% for < 30 years; 3.6% for ≥ 60 years). Prevalence of HBsAg was 0.8-0.9% below the age of 10 years, and higher but similar in older age groups (2.3-2.7%). There was an association between literacy and prevalence of hepatitis, particularly for HCV. Hospital admission, surgical operation, blood transfusion, and intravenous drug use were the main risk factors, and they were associated independently with a higher prevalence rate of viral hepatitis.

**Conclusions:**

Libya may be considered an area of low-intermediate endemicity for hepatitis B virus infection, with lower rates in young age groups, and an area of low endemicity for hepatitis C. The prevalence of hepatitis B and C across Libya is not homogeneous, with indications of the effect of the higher rates in some neighbouring countries. Libya should adopt full coverage national plans and guidelines to face the future consequences of viral hepatitis, particularly hepatitis C virus.

## Background

Hepatitis B and C viruses (HBV and HCV) are the major causes of liver diseases in the world. The relative importance of HBV and HCV infections varies greatly from one part of the world to another and changes over time [1]. Worldwide, over two billion people are infected by HBV alone, of whom about one million die annually. Hepatitis C virus affects about 200 million people [2]. However, in many countries its prevalence is expected to surpass that of hepatitis B, particularly among certain risk groups that are vulnerable to both hepatitis B and C viruses [3].

The endemicity of HBV infection varies greatly over the world, from highly endemic areas (> 8% infection rate), to intermediate (2-8%) and low endemicity areas (< 2%). Africa is among the highly endemic areas, but some countries in the north fall in the intermediate category, with an average rate of about 7%, whereas most regions of west and east Africa are highly endemic areas with chronic infection rates of 7-10% [4]. In some countries, such as Senegal, a large part of the population will be exposed in the course of their lives to HBV and become infected [5]. All of central and southern Africa is also in the high endemicity category [6]. Few studies evaluated the status of HBV in Libya. In 2000, Daw et al. [7] reported a HBV prevalence of 10% among hospital health-care workers and that infection was associated with certain occupational risk factors.

Hepatitis C virus infection is a major public health problem [8,9]. African countries have among the highest prevalence rates of HCV in the world, ranging from 1 to 26% [10,11]. More than 28 million people are chronically infected with HCV in this continent, and it is difficult to speculate about current and future trends [3,12]. In Libya, one study reported that the prevalence of HCV among different populations varied according to the risk factor involved [13]. Recently, a comprehensive study was carried out on HCV genotypes in Libya. Hepatitis C virus genotype 4 was the predominant one, followed by HCV genotype 1 and then other less common genotypes [14]. However, further studies are needed to clarify the magnitude and impact of HCV in Libya.

Viral hepatitis has tremendous socioeconomic, health-care and even political repercussions. A better understanding of the epidemiology of viral hepatitis and the risk factors involved is among the priorities of any nation [15,16]. Reliable epidemiological data on prevalence rates and transmission routes [17,18] of viral hepatitis are essential for designing national control policies. The epidemiological data in Africa are scanty and vary greatly from one region to another [4]. Therefore, each country should adopt its own strategy to combat viral hepatitis.

Libya has an area of 1,775,500 km^2^ and a population reported in mid 2006 as 5,323,991, giving a population density of 2.9 persons per km^2^. The country boasts the highest literacy and educational enrolment in North Africa and among the Arab nations [19,20]. Life expectancy (73 years) and health-adjusted life expectancy (64 years) are among the best in the Middle East and North Africa [19,21]. Nevertheless, Libyan health services have been hampered by bureaucracy and lack of proper long-term planning [20,22]. Both Libyan and international medical experts have voiced concerns about the potential for increases in infection, particularly viral hepatitis and AIDS [23,24].

Recent data on the prevalence of hepatitis B and C viruses and risk factors among the Libyan population are lacking. Such data are important for understanding the burden of viral infection and for predicting future trends. Implementation of surveillance to guide public health policy is needed to efficiently control viral hepatitis spread in this country, and this requires reliable epidemiological data. In this comprehensive nationwide study we determined the prevalence of hepatitis B and C virus infections among the Libyan population and analysed the risk factors.

## Methods

### Study population

We conducted a national cross-sectional study stratified by region and age. The sample size in each region was set ahead of the survey and recruitment continued until the sampling teams fulfilled the required sample size. Those enrolled from each region were selected randomly. Regional representativeness was fulfilled by classifying the country into 12 regions including all administrative divisions and sampling based on population proportions. Sample sizes of males, females and age strata were determined by multi-stage cluster sampling in each region proportional to regional populations according to the latest national census in 2003–2004. Teams of medical doctors and nurses established the sampling frames and collected the information and samples in all the regions as described in Table 1. The following targeted age strata were selected:

– < 6 years: Pre-school children (maternal and child care centres);

– 7–19 years: School age below university level (primary, preparatory and high schools);

– 20–49 years: University graduate and post graduate students (universities and health services centres);

– ≥ 50 years: Employed and retired personnel (different community and governmental sectors).

**Table 1:** Numbers of samples collected from the 12 Libyan regions

**Region**	**Number**	**Percent**	Ratio*
Albatnan	4996	7.6	1.80
Aljabal Alakhdar	5944	9.0	1.10
Benghazi	6938	10.6	1.10
Ajdabia	4963	7.5	1.10
Sirt	3871	5.9	1.75
Misrata	4966	7.6	1.90
Almergeb	4913	7.5	1.10
Tarhuna	5023	7.6	1.10
Tripoli	8013	12.2	1.10
Alzawia	5022	7.6	1.80
West mountain	5752	8.7	1.10
Fezzan	5360	8.2	1.90
Total sample	65761	100	1.10

*Ratio of individuals testing positive for hepatitis B and C viruses to the total population in each region.

### Demographic and epidemiological data questionnaire

Sample size calculations were based on the prevalence of HCV and HBV infections in prior studies in Libya. To detect a two-fold increase in prevalence among the lowest prevalence expected for HCV 1/1000 in each age group as compared with the next age, a sample of 25,476 has a power of 80% (beta error <0.2) to detect this difference at the alpha = 0.05 level (one-sided). A total sample of 51,000 would allow estimation of these parameters independently for males and females.

Individuals confirmed to be infected with HCV were referred to the main teaching hospitals in the major regions: Tripoli Central Hospital and Tripoli Medical Centre for western and central regions, Benghazi Medical Centre for the eastern regions, and Sebha Medical Centre for Fezzan area. These hospitals receive all individuals who test positive for anti-HCV, HBV or HIV from all over the country as a policy run under the control of the Libyan National Health Authority.

The questionnaire sought information about age, gender, education, residence and occupation, as well as history of major invasive interventions, use of recreational intravenous drugs, and promiscuous sexual practices. The questionnaire was completed by each participant and those of a younger age were helped by their parents in the presence of medical doctors and social assistants, who recorded the needed information and coded the questionnaire.

### Laboratory tests

A blood sample of 5-10 ml was collected and transported immediately to the local laboratory in a general hospital within the same region. Serum was prepared and stored at -20°C until tested as previously described [14].

All samples were tested for HBsAg and anti-HCV markers by using a third-generation enzyme immunoassay (Axsym; HCV EIA 3.0; Abbott Laboratories, Abbott Park, Illinois, and HBsAg, Axsym) as previously published [14]. Samples that were positive for anti-HCV were retested by the same method, and only if the retest was positive were they considered as positive.

### Statistical analysis

Data were coded and entered into a data base, which was then cleaned and verified. Data were analysed by using the Chi-square test with Yates' correction or Student's *t*-test for univariate analysis. Multivariate analysis was performed by using logistic regression, with anti-HCV serologic results as the dependent variable (SPSS, Inc., Chicago, Illinois). Prevalence estimates are reported with 95% confidence intervals (CI) determined by using the Poisson distribution approximation [18]. A type I error of α = 0.05 was assumed. Sample weighting was later performed and a weight was calculated according to age and sex within each region.

### Ethical considerations

The study was approved by the Libyan National Ethical Committee (Approval No. LY NS; HV299789). It was conducted in accordance with the Helsinki Declaration [25] and under the supervision of the Libyan Centre for Disease Control. All participants signed an informed consent form witnessed by the local health office before collection of data and blood samples. The questionnaire used to collect demographic and epidemiological data (Additional file 1: Table S1) was anonymous and linked to the blood sample tube only by a code.

## Results

**Figure 1:**
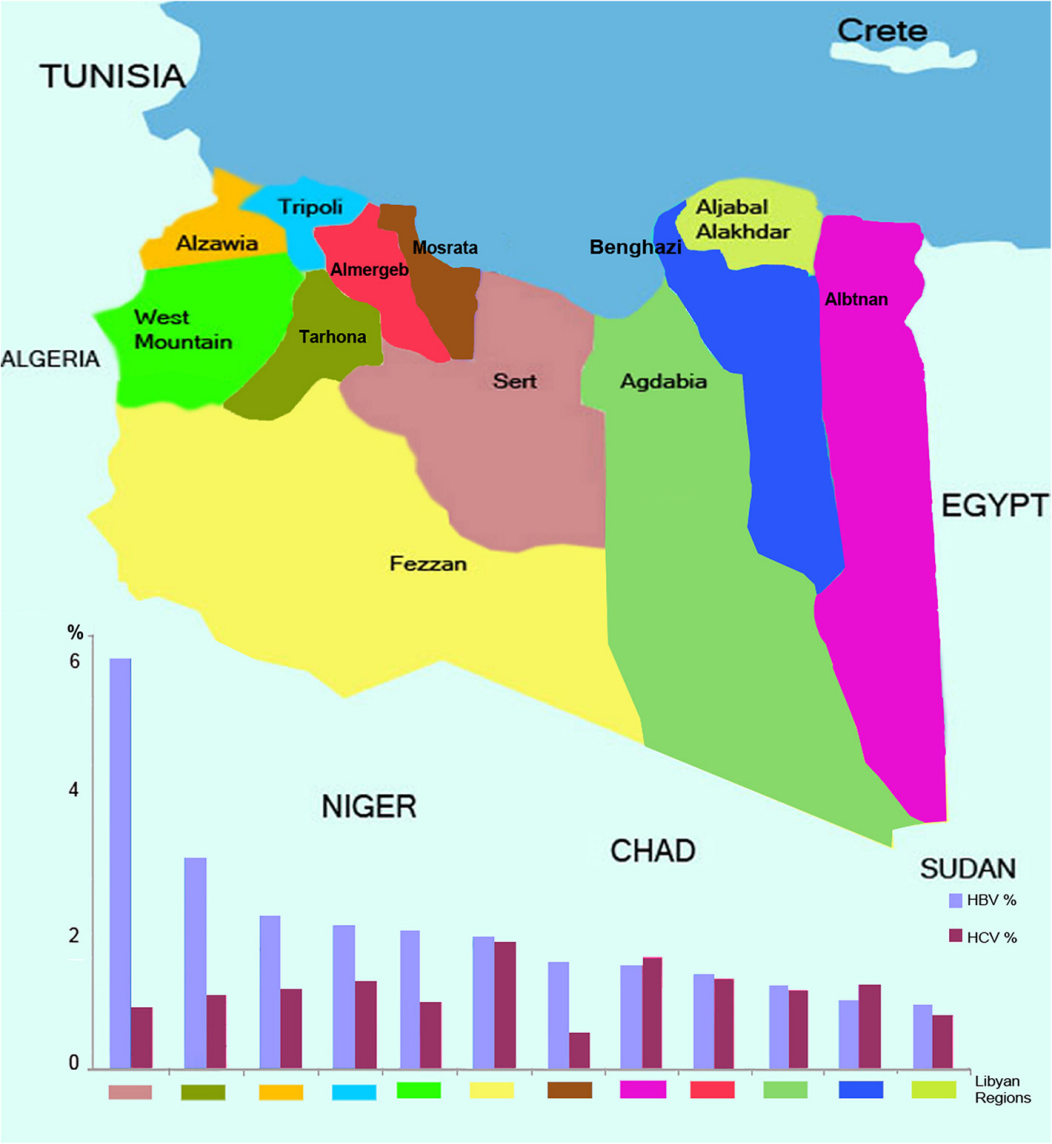
Map of Libya showing the prevalence of hepatitis B and C infections by region.

The overall prevalence of HBsAg positivity was 2.2% (95% CI 2.1%-2.3%). The prevalence varied from one region to another (Figure 1). The rate was highest in Sirt (6.6%) and Tarhuna (3.4%), and lowest in Aljabel Alakhdar (1.0%), Benghazi (1.0%), and Ajdabia (1.4%). The overall prevalence of HCV was 1.2% but it varied from one region to another (Figure 1). The highest rates were in Fezzan (2.2%) and Albatnan (1.8%) and the lowest were in Misrata (0.6%), Sirt (1.0%) and Western Mountain (1.1%). The rate of HBV prevalence was significantly higher in males (2.6%; 95% CI 2.4-2.7) than in females (1.8%; 95% CI 1.6-1.9). The probability of being HBsAg positive was 1.4 times higher among males than females.

The overall unweighted prevalence of HCV was 1.2% (95% CI 1.1-1.3), and it rose to 1.3% after weighting. For those aged > 20 years, the unweighted and weighted percentages were not significantly different (1.5% and 1.6%, respectively). The prevalence rate was slightly higher among females than males but the difference was not statistically significant. The prevalence rates weighted for other factors (age group, education, marital status, type of dwelling, and risk exposure) are shown in Additional file 2: Table S2.

**Figure 2:**
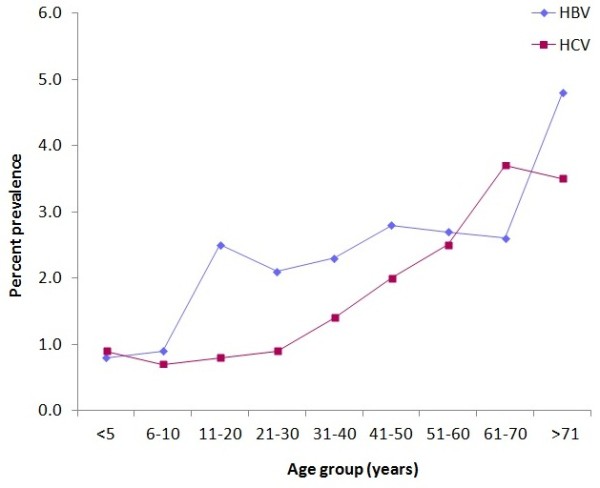
Prevalence of hepatitis B and C infections by age, Libya, 2008.

Figure 2 shows the prevalence of HBV and HCV among the Libyan population according to age. The prevalence of anti-HCV was stable at 0.8-0.9% until the age of 30 years, after which it rose steadily, reaching 2.7% in those above the age of 50 years. In contrast, the prevalence of HBsAg was lowest in the youngest age group (0.9%), and in the older groups it fluctuated between 2.0% and 2.9% without any evident temporal trend. However, it rose sharply after the age of 70 years.

The overall prevalence rates of HCV in males and females were similar (1.1% and 1.3%, respectively). The mean ages of anti-HCV positive females (31.7 ± 18.4 years) and males (35.6 + 20.9 years) were significantly higher (p < 0.001) than those of HBsAg positive females (26.3 + 16.8 years) and males (30.0 + 17.6 years). The mean age of HCV positive individuals was significantly higher than that of anti-HCV negative individuals for both males (almost 10 years difference) and females (7 years difference). The mean age of HBsAg positive individuals was also significantly higher (but to a lesser degree) among females (by < 2 years) and males (by < 4 years). More than 50% of the HBsAg positive individuals and about 40% of HCV positive cases were below 30 years of age.

**Figure 3:**
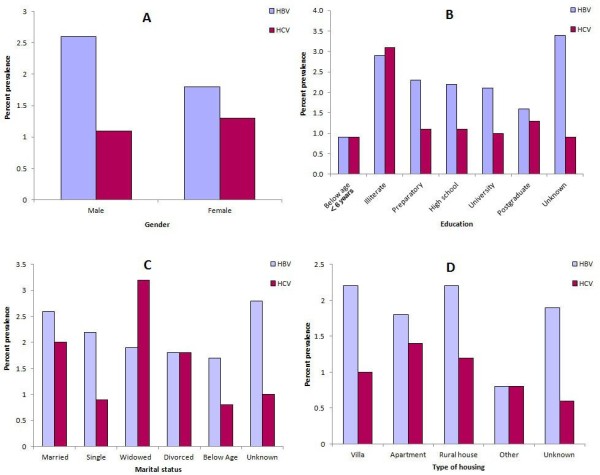
**Prevalence of hepatitis B and C infections by demographic and social variables, Libya, 2008.A**. Gender **B**. Education **C**. Marital Status **D**. Type of housing.

The associations between hepatitis B and C virus infections and various factors are shown in Table 2. Hepatitis B virus was more prevalent among the illiterate group (2.9%) than among the literate groups: 1.6% among postgraduates and 2.3% among preparatory school students. The prevalence of HCV was also higher among the illiterate group (3.1%) whereas in the literate groups it ranged from 0.9% to 1.1%. The influence of marital status and living standard on the prevalence of hepatitis B and C is shown in Figure 3. Hepatitis B was more prevalent among the widowed, whereas HCV was more prevalent among single and younger individuals.

**Table 2:** Association between hepatitis B and C virus infections and influencing factors among the Libyan population

	**HCV**				**HBsAg**			
		**OR**	**95.0% C.I.**			**OR**	**95.0% C.I.**	
			**Lower**	**Upper**			**Lower**	**Upper**
Age group (years) relative to < 6								
6-10		0.74	0.46	1.18		1.21	0.79	1.86
11-20		0.91	0.67	1.24		3.36	2.5	4.52
21-30		0.88	0.63	1.22		3.01	2.21	4.1
31-40		1.41	1.01	1.96		3.14	2.28	4.32
41-50		1.87	1.32	2.64		3.77	2.7	5.26
51-60		2.36	1.65	3.37		3.7	2.59	5.28
61-70		3.27	2.17	4.93		3.34	2.13	5.24
>70		3.32	2.01	5.49		5.69	3.54	9.15
Males vs females		0.86	0.74	0.99		1.51	1.35	1.68
Hospital admission		1.32	1.09	1.61				
Dental procedure		0.84	0.71	0.99		0.85	0.76	0.96
Blood transfusion		1.55	1.22	1.97				
Intravenous drug use		6.41	2.27	18.07		0	0	-
Surgery		1.35	1.08	1.67				
Family HBV		1.52	1	2.3		1.97	1.46	2.64
Haemodialysis		4.37	1.71	11.2				
Contact with HBV						1.66	1.23	2.23
Skin piercing						1.36	1	1.83

**Figure 4:**
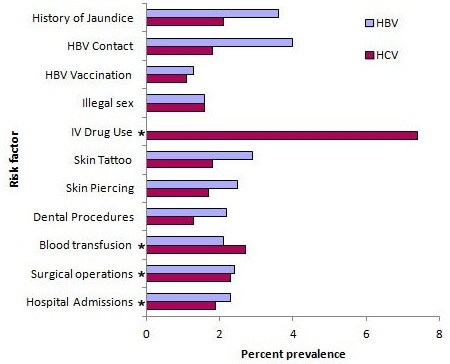
Prevalence of hepatitis B and C infections by risk factors, Libya, 2008 *Statistically significant at 0.05.

We also analysed the influence of risk factors, such as hospital admission, surgical operation, blood transfusion, intravenous drug use, and sexual behaviour, on the prevalence of hepatitis B and hepatitis C (Figure 4). Hepatitis B virus was most prevalent among those with previous surgical operations (2.4%) or history of hospital admission (2.4%), followed by blood transfusion (2.1%) and promiscuous sexual behaviour (1.6%). Hepatitis C was most prevalent among intravenous drug users (7.4%) and less prevalent but still substantial in those undergoing blood transfusion (2.7%), surgical operation (2.3%) or hospital admission (1.9%).

Table 3 shows the distribution of the estimated seroprevalence of HBV and HCV according to demographic characteristics. There were minimal differences in the estimated number of cases between males and females for HCV, but more than 60% of the estimated number of HBsAg positive individuals were males. Over 50% of them were below 30 years old, and about 40% of HCV cases were also below that age. In terms of educational level, the highest prevalence rate of infection was among illiterates for both viruses (weighted prevalence 2.6% for HBsAg, representing 12.3% of all estimated cases, and 3.0% for HCV, representing 21.8% of all estimated cases).

**Table 3:** Number of persons chronically infected by HBV and of anti-HCV positive individuals in Libya according to age, socio-demographic factors and risk factors

	**Nationwide estimated number of HBsAg positive persons**		**Nationwide estimated number of anti-HCV positive persons**	
	**n**	**%**	**n**	**%**
**Total**	107,848	100	68,275	100.0
**Gender**				
Females	42,297	39.2	34,845	51.0
Males	65,551	60.8	33,430	49.0
**Age groups**				
≤ 5	4,861	4.5	4,699	6.9
6-10	4,110	3.8	3,881	5.7
11-20	26,148	24.2	8,889	13.0
21-30	26,153	24.2	10,147	14.9
31-40	20,139	18.7	13,966	20.5
41-50	11,436	10.6	9,738	14.3
51-60	6,856	6.4	6,633	9.7
61-70	3,613	3.4	6,415	9.4
≥71	4,532	4.2	3,907	5.7
**Education**				
Below school age	4,957	4.6	5,235	7.7
Illiterate	13,318	12.3	14,850	21.8
Preparatory	34,927	32.4	20,685	30.3
High school	34,389	31.9	17,214	25.2
University	16,118	14.9	7,544	11.0
Post graduate	1,769	1.6	981	1.4
Unknown	2,370	2.2	1,766	2.6
**Marital status**				
Married	39,844	36.9	32,605	47.8
Single	42,551	39.5	17,845	26.1
Widowed	1,425	1.3	3,403	5.0
Divorced	913	0.8	470	0.7
Below age	21,434	19.9	12,828	18.8
Unknown	1,681	1.6	1,124	1.6
**Type of dwelling**				
Villa	13,545	12.6	6,944	10.2
Apartment	10,491	9.7	10,480	15.3
Rural house	80,646	74.8	49,398	72.4
Other	118	0.1	104	0.2
Unknown	3,048	2.8	1,349	2.0

## Discussion

The few studies on the prevalence of hepatitis B and C viruses in Libya examined specific population groups, such as parturient women and their newborns [26], healthcare workers, blood donors, transfusion patients [13] and hemodialysis patients [27], or other issues such as prevalence of genotypes [14]. However, none of these studies lend themselves to extrapolation to the general population, and our study, the first of its kind in Libya and the largest in Africa, was done to provide a basis for prioritising public health measures in Libya.

In the European Mediterranean countries, the overall prevalence of HBV ranges between 2.5% and 3.5% [28,29]. In contrast, North Africa is overall an area of relatively high HBV endemicity. Tunisia, Algeria and Morocco fall in the intermediate category, with current infection rates of about 7% [30]. But Egypt is classified among the highest endemicity countries, along with Sudan, Chad and Niger, all of which neighbour Libya [31,32]. The lower prevalence rate of HBV we observed (2.2%) classifies Libya in the lower part of the low-intermediate endemicity class (2-7% category). This lower rate could be due to the better socioeconomic conditions and the early efforts by the National Prevention Program of Infectious Diseases in Libya. Vaccination against HBV infection has been strongly encouraged and offered free of charge in Libya since 1989, and in 1991 it became compulsory for infants 3 months of age and children 12 years of age.

Hepatitis C is an emerging problem among the Mediterranean countries, where the estimated prevalence ranges from 1.0-2.6%, and isolated areas in Italy and Greece have rates of 7-20% among the general adult population [33,34]. A similar picture was also found in Tunisia and Morocco, where the rates ranged from 1.7 to 2.9%, with higher rates in some regions [35,36]. Hepatitis C virus screening and surveillance is minimal at the national level in these countries. In Libya, a study conducted between 1991 and 2001 indicated that the prevalence of HCV ranged from 1.2% to 1.6% among blood donors and healthy individuals but was much higher (20.5%) among hospital personnel [13]. The 1.2% rate we observed in our country-wide study classifies Libya as a low endemicity country. Libya has made screening mandatory for all blood donors, pregnant women, and patients at high risk of having surgery, and has introduced a national registry system covering all positive individuals.

We were not surprised by the substantial regional variation in prevalence rate in Libya. Such variation has also been reported in other Mediterranean countries, such as Italy, Greece and Spain [37-39]. The variation observed in Libya is probably related to different factors, including socioeconomic conditions, but one particular observation is noteworthy. The prevalence of HCV was highest in the southern region of Fezzan (2.08%) and the eastern region of Albatnan (1.77%). The high prevalence in the southern region could be related to its proximity to sub-Saharan countries and the hosting of many African immigrants. Likewise, the eastern region of Albatnan borders Egypt, which has the highest prevalence rate of HCV in the world [40,41], and from where many workers, both legitimate and illegal, come to Libya.

In Libya, the prevalence of HBV was low in children < 10 years of age (0.8%), and in the other age groups it ranged from 2.0 to 2.9%. In most other African countries the prevalence is high among infants and increases rapidly until the age of 30 years, and it rises even higher among those over forty years old [5,6,42]. The lower rate in the Libyan population could be due to the compulsory children vaccination program, which was introduced earlier in Libya than in other African countries.

The overall prevalence of HCV in our study was not significantly different between females and males, and it increased gradually after the age of 30 years. This is in agreement with previous studies carried out in Libya, which showed a peak in the prevalence of HCV among those 46-55 years old [13]. Other studies, particularly from North America, also showed the highest prevalence in the 30-49 year age group in all racial groups [43,44].

We also analysed the risk factors associated with HBV and HCV infections. The influence of risk factors on the prevalence rate was evident for HCV. The prevalence rate among those with risk factors ranged from 1.9 to 7.4%, whereas among those without risk factors it was 1.3% (*p* < 0.025). The most prominent risk factor was intravenous drug use, with 7.4% of intravenous drug users testing positive for HCV. In this context, it is important to note that active drug users are less likely to participate in health surveys, and the actual overall prevalence rate is likely higher than the observed rate [45,46]. A recent study on ~1200 Libyan patients chronically infected with HCV correlated genotypes with risk factors. That study indicated that recent drug use was significantly linked to emergence of new genotypes, lending further credence to the role of intravenous drug use in HCV transmission in Libya [13]. Accordingly, we expect that the prevalence of HCV may increase in Libya because there is consensus that HCV-infected populations are heavily weighted towards intravenous drug use worldwide [47-49].

Our findings show that the prevalence rates of HBV and HCV in Libya are relatively low, and this could be an indicator of the success of the national efforts to control these infections. However, we only assessed a marker of the prevalence of HBV chronic infection (HBsAg), which represents the reservoir of infection and of future complications (cirrhosis and liver cancer). We did not estimate the proportion of those who had ever been infected in Libya, which can be estimated by testing for serum anti-HBc antibodies. Moreover, the higher rates in regions bordering high endemicity countries and the poorly controlled state of these borders can lead to an increase in prevalence, especially during this time when the country as whole is experiencing many difficulties. Continuous surveillance and maintenance of the national program for combating hepatitis are essential, and further studies are needed, particularly on co-infection with HIV, which is a problem in this region. Our findings would be useful for making estimates and projections about the overall disease burden, including the complications of hepatitis infection, such as cirrhosis, liver failure, and hepatocellular carcinoma [50]. Recently, El-Bouzedi [51] constructed a mathematical model using published epidemiological data on viral hepatitis to estimate the future consequences of hepatitis infection in Libya. Such modelling will inform health policy, resource allocation and healthcare delivery and may improve the management of patients with viral hepatitis.

## Competing interests

The authors state that they have no competing interests.

## Authors’ contributions

MD designed the study, extracted the data, and drafted and finalised the manuscript. AE analyzed the data and contributed to the drafting of the data. Both authors read and approved the final manuscript.

## Pre-publication history

The pre-publication history for this paper can be accessed here:

http://www.biomedcentral.com/1471-2334/14/17/prepub

## Supplementary Material

Additional file 1: Table S1 HBV, HCV and HIV questionnaire form used to collect the data from Libyan populations. Click here for file

Additional file 2: Table S2 Sero-prevalence of HBsAg and anti-HCV antibodies among Libyans according to demographic characteristics and risk exposures. Click here for file
