# Expression of concern: Prevalence of hepatitis B and hepatitis C infection in Libya: results from a national population based survey

**DOI:** 10.1186/s12879-015-0772-8

**Published:** 2015-02-19

**Authors:** Philippa K Harris

**Affiliations:** BioMed Central, 236 Gray’s Inn Road, London, WC1X 8HB UK

Following publication of this article (Daw and El-Bouzedi, BMC Infectious Diseases 2014, **14**:17) [1] it has been brought to the Editors’ attention that much of this data appears to have been previously published in the following article [2]. In addition concerns about the authors’ right to use this data have been raised. Despite extensive efforts to obtain an independent investigation, we have been unable to secure advice on the matter. Given the uncertainty, the Editors advise the readers to interpret the data with due caution. We apologize to all affected parties.
