# Supplementary material for: Prevalence of hepatitis B and hepatitis C infection in Libya: results from a national population based survey
Source: BMC Infect Dis. 2014 Jan 9;14:17. doi: 10.1186/1471-2334-14-17 (PMC3893419; doi:10.1186/1471-2334-14-17)
Supplement: Additional file 1: Table S1 — HBV, HCV and HIV questionnaire form used to collect the data from Libyan populations. [file 1471-2334-14-17-S1.doc]

**HBV, HCV and HIV Questionnaire**

**Constructed by Mohamed A Daw and Abdallah El-Bouzedi ; Approved by National Ethical Committee**

**-------------------------------------------------------------------------------**

|  |  |  |  |  |  |
| --- | --- | --- | --- | --- | --- |

**Questionnaire Identification Number:**

**----------------------------------------------------------------------------------------------------------------------------**

**DEMOGRAPHIC INFORMATION: (check all that apply and put cross in the box)**

**Address:** …………………………………………………………………………………….

**Date of birth:** ……**/**……..**/**…….. **Age (years):** ………………… **Sex: Male 🞏 Female 🞏**

**Place of birth:** Country………………City …………………County

**Place of living** (specify the name) City: ………………………..….. Small city: …………....…………..

Village: ………………………………..Countryside………………………….

**Area name and code**: ……………………………….

**What kind of whom do you live:** Council house 🞏 Private flat 🞏 Private house 🞏

**How do you earn money for life?** Relay on family 🞏 working in the summer 🞏

**In your opinion is your family standard of living?** V. Good 🞏 Good 🞏 Normal/average 🞏 poor 🞏 V. poor 🞏

**Total Family member:** ………… Male: ……………Female: …………..

**Name of school/Collage attend:** Primary**:** ......................................... Secondary: ……….…………….. Higher School ……………….……………………… Department: Science 🞏 Education 🞏

Collage: ………………………..…………….……Specify subject: ………………………………….

Grad average: ……………………………………………

**Classify the area of living:** V. Good 🞏 Good 🞏 Normal/average 🞏 poor 🞏 V. poor 🞏

**Total Family member:** ………… Male: ……………Female: …………..

**Father age**: …………………...level of education: .……………………... occupation: ………......

Monthly income: …………………..

**Mother age:** …………….level of education: ……………….………………occupation: …………..

Monthly income: ……………………..

**What is your parent’s marital status?** Married 🞏 widowed 🞏 Divorced

**Specify Your Family income?** 150-300 🞏 300-450 🞏 450-600 🞏 600-750 🞏 750-900 🞏 900-1050 🞏 1050-1200 🞏 1200-1350 🞏 1350-1500 🞏 >1500 🞏

**In your opinion is your family income?** M. Enough 🞏 V. Enough 🞏 Enough 🞏 Normal/average 🞏 N. Enough 🞏

**CLINICAL DATA/SYMPOMS:**

**Did the patient experience? (Answer for each symptom below)**

**Abdominal Pain:** Yes 🞏 No 🞏 Unknown 🞏

**Dark Urine:** Yes 🞏 No 🞏 Unknown 🞏

**Diarrhea:** Yes 🞏 No 🞏 Unknown 🞏

**Fatigue:** Yes 🞏 No 🞏 Unknown 🞏

**Jaundice:** Yes 🞏 No 🞏 Unknown 🞏

**Nausea:** Yes 🞏 No 🞏 Unknown 🞏

**Was the patient hospitalized for hepatitis?** Yes 🞏 No 🞏 Unknown 🞏

**VACCINATION HISTORY: (Put cross in the box below)**

**Have you ever received the hepatitis A vaccine?** Yes 🞏 No 🞏 Unk 🞏

**If yes,** how many doses? One 🞏 ≥ 2 🞏

**In what year was the last dose received?** ..........................................................................

**Have you ever received the hepatitis B vaccine?** Yes 🞏 No 🞏 Unk 🞏

**If yes, how many doses?** 1 🞏 2 🞏 3+🞏

**In what year was the last dose received?** ..........................................................................

**PATIENT INFORMATION/HISTORY: (Put cross in the box below)**

**In general, would you say your health is?** Excellent 🞏 Very good 🞏 Fair 🞏 poor 🞏 v. poor 🞏

**Compared to one year ago, how would you rate your health in general now?**

Much better now than one year ago 🞏 Somewhat better now than one year ago 🞏 About the same as one year ago 🞏 Somewhat worse than one year ago 🞏 Much worse than one year ago 🞏

**Do you smoke?** Yes 🞏 No 🞏 **If yeas,** how many cigarettes a day: 5-10 🞏 11-16 🞏 17-22 🞏 23-28 🞏 29-34 🞏 35 -40 🞏

**Are you diabetic?** Yes 🞏 No 🞏

**Do you drink alcohol?** Yes 🞏 No 🞏 **If yeas,** how many time a week: 1🞏 2 🞏 3 🞏 4 🞏 > 4🞏

**Have you ever been tested for the following?** Please put a cross in each box that applies. HIV/AIDS 🞏 HBV 🞏 HCV🞏

**Do you take drugs?** Yes 🞏 No 🞏 **If yeas,** specified: Heroin 🞏 Cocaine 🞏 ……………..

**During the 2 weeks-6 months, have you been in contact with a person with confirmed or suspected acute or chronic hepatitis B/C / HIV?** Yes 🞏 No 🞏 Unk 🞏

**In the 6 months before symptom onset,**

**(Ask both of the following questions regardless of the patient’s gender)**

**How many male sex partners did the patient have?** 0 🞏 1 🞏 2-4 🞏 >4 🞏 Unk 🞏

**How many female sex partners did the patient have?** 0 🞏 1 🞏 2-4 🞏 >4 🞏 Unk 🞏

**Have you ever treated for a sexually transmitted disease?** Yes 🞏 No 🞏 Unk 🞏.

**If yes,** which disease(s): ………………………………………

**What was the most recent treatment?** 6 month: 🞏 One year 🞏 year and half 🞏 Two year 🞏

**Have you had sex without using condom?** Yes 🞏 No 🞏 Unk 🞏

**Have you had sexual contact with someone who has had sex with someone other than you?**

Yes 🞏 No 🞏 Unk 🞏

**Do you take care about using condoms?** Yes 🞏 No 🞏 Unk 🞏

**Do you use condoms for oral sex?** Yes 🞏 No 🞏 Unk 🞏

**Where do you get information on HIV/HCV or other sexually Transmitted infection? Please put a cross in each box that applies?** Family 🞏 Friends/peers 🞏 Media 🞏 School 🞏 Social/ health workers 🞏 Other place 🞏 (please specify) ………………………No place-I don’t get information on HIV/HCV 🞏

**During the 2 weeks -6 months prior to onset of symptoms**

**Did you inject drugs not prescribed by doctor?** Yes 🞏 No 🞏 Unk 🞏

**Did you use street drugs (not injected)?** Yes 🞏 No 🞏 Unk 🞏

**Undergo hemodilysis?** Yes 🞏 No 🞏 Unk 🞏

**If yes,** month and year of hemodialysis: …………………………………………………

**Did you have an accidental stick or puncture with a needle or other object contaminated with blood?** Yes 🞏 No 🞏 Unk 🞏

**Did you receive blood products [transfusion]?** Yes 🞏 No 🞏 Unk 🞏

**Did you receive any outpatient IV infusions and /or injection?** Yes 🞏 No 🞏 Unk 🞏

D**id you ever inject drugs?** Yes 🞏 No 🞏 Unk 🞏

**Did you have other exposure to someone else’s blood?** Yes 🞏 No 🞏 Unk 🞏

**Did you receive a tattoo?** Yes 🞏 No 🞏 Unk 🞏

**Did you have any part of your body pierced (other than ear)?** Yes 🞏 No 🞏 Unk 🞏

**Did you have dental work or oral surgery?** Yes 🞏 No 🞏 Unk 🞏

**Did you have surgery (other than oral)?** Yes 🞏 No 🞏 Unk 🞏

**Were you hospitalized?** Yes 🞏 No 🞏 Unk 🞏

**Were you incarcerated for longer than 24 hours?** Yes 🞏 No 🞏 Unk 🞏

**During your lifetime, were you ever incarcerated for longer than 6 months?** Yes 🞏 No 🞏 Unk 🞏

**Do you use the internet on the computer?** Yes 🞏 No 🞏

**What organizations do you know of that provide HIV/HCV help?** ..........................

**Are you satisfied with the work of that organization?** Yes 🞏 No 🞏 Unk 🞏

**If you needed HIV/HCV help, who would you go to?** ..................................................

**What kind of services are missing for young gay population**?

**Please state how think the interview went:**

Very well 🞏 moderately 🞏 not very well 🞏

**If not very well please state why. Add any other comments you might have**

**DIAGNOSTIC TESTS:**

**HCV serology results: start and tick box below (check all that apply)**

**Date when (1^st^) blood drawn for hepatitis C testing** .........**/**……**/**…….

**Reporting Laboratory:** ………………………………………………………………

**Antibody to hepatitis C virus [anti-HCV]**

Positive 🞏 Negative 🞏 Unknown 🞏 Not done 🞏

**anti-HCV signal to cut-off ratio**……………………………………………………...

**HCV RNA [e.g., PCR]**

Positive 🞏 Negative 🞏 Unknown 🞏 Not done 🞏

**Liver enzyme values:**

**SGPT (ALT)**……..………Test date:……/……/……..Upper limit normal: …………...

**SGOT(AST)**……………..Test date: …../……/……...Upper limit normal:…………...

**Other tests…**……………………………………………………………………..…….
