# Supplementary material for: Prevalence of hepatitis B and hepatitis C infection in Libya: results from a national population based survey
Source: BMC Infect Dis. 2014 Jan 9;14:17. doi: 10.1186/1471-2334-14-17 (PMC3893419; doi:10.1186/1471-2334-14-17)
Supplement: Additional file 2: Table S2 — Sero-prevalence of HBsAg and anti-HCV antibodies among Libyans according to demographic characteristics and risk exposures. [file 1471-2334-14-17-S2.docx]

**Supplemental Table S2**: Sero-prevalence of HBsAg and anti-HCV antibodies among Libyans according to demographic characteristics and risk exposures.

|  |  | HBsAg Positive | | | Anti-HCV Positive | | | Sig. | |
| --- | --- | --- | --- | --- | --- | --- | --- | --- | --- |
|  | Total ‘n’ | Count | Sample  % | Weighted  % | Count | Sample  % | Weighted  % | HBsAg | HCV |
| **Total** | 65,761 | 1,431 | 2.2 | 2.0 | 780 | 1.2 | 1.3 |  |  |
| **Gender** |  |  |  |  |  |  |  |  |  |
| Females | 32,996 | 591 | 1.8 | 1.6 | 413 | 1.3 | 1.3 | .000 | 0.119 |
| Males | 32,765 | 840 | 2.6 | 2.4 | 367 | 1.1 | 1.2 |  |  |
| **Age group** |  |  |  |  |  |  |  |  |  |
| < 5 | 6,530 | 55 | 0.8 | 0.9 | 57 | 0.9 | 0.8 |  |  |
| 6 -10 | 4,091 | 38 | 0.9 | 0.8 | 28 | 0.7 | 0.7 |  |  |
| 11-20 | 20,658 | 520 | 2.5 | 2.3 | 168 | 0.8 | 0.8 |  |  |
| 21-30 | 13,883 | 289 | 2.1 | 2.3 | 119 | 0.9 | 0.9 |  |  |
| 31-40 | 9,775 | 220 | 2.3 | 2.3 | 140 | 1.4 | 1.6 |  |  |
| 41-50 | 5,359 | 150 | 2.8 | 2.4 | 108 | 2.0 | 2.0 |  |  |
| 51-60 | 3,427 | 91 | 2.7 | 2.7 | 86 | 2.5 | 2.6 |  |  |
| 61-70 | 1,344 | 35 | 2.6 | 2.1 | 50 | 3.7 | 3.7 |  |  |
| >70 | 694 | 33 | 4.8 | 3.1 | 24 | 3.5 | 2.7 | .000 | .000 |
| **Education** |  |  |  |  |  |  |  |  |  |
| Below age | 6,485 | 56 | 0.9 | 0.9 | 60 | 0.9 | 0.9 |  |  |
| Illiterate | 5,084 | 146 | 2.9 | 2.6 | 156 | 3.1 | 3.0 |  |  |
| Preparatory | 22,431 | 520 | 2.3 | 2.0 | 236 | 1.1 | 1.2 |  |  |
| High School | 20,646 | 458 | 2.2 | 2.3 | 212 | 1.0 | 1.1 |  |  |
| University | 8,592 | 184 | 2.1 | 2.2 | 90 | 1.0 | 1.0 |  |  |
| Post Graduate | 1,007 | 16 | 1.6 | 1.9 | 13 | 1.3 | 1.0 |  |  |
| Unknown | 1,516 | 51 | 3.4 | 2.0 | 13 | 0.9 | 1.3 | .000 | .000 |
| **Marital status** |  |  |  |  |  |  |  |  |  |
| Married | 17,862 | 472 | 2.6 | 2.5 | 351 | 2.0 | 2.1 |  |  |
| Single | 26,634 | 576 | 2.2 | 2.2 | 230 | 0.9 | 0.9 |  |  |
| Widowed | 808 | 15 | 1.9 | 1.4 | 26 | 3.2 | 3.4 |  |  |
| Divorced | 337 | 6 | 1.8 | 2.9 | 6 | 1.8 | 1.5 |  |  |
| Below Age | 18,994 | 330 | 1.7 | 1.4 | 156 | 0.8 | 0.8 |  |  |
| Unknown | 1,126 | 32 | 2.8 | 2.1 | 11 | 1.0 | 1.4 | .000 | .000 |
| **Type of dwelling** |  |  |  |  |  |  |  |  |  |
| Vila | 9,998 | 218 | 2.2 | 2.2 | 96 | 1.0 | 1.2 |  |  |
| Apartment | 7,350 | 135 | 1.8 | 1.6 | 103 | 1.4 | 1.6 |  |  |
| Rural house | 46,088 | 1,035 | 2.2 | 2.1 | 567 | 1.2 | 1.3 |  |  |
| Other | 120 | 1 | 0.8 | 1.1 | 1 | 0.8 | 1.0 |  |  |
| Unknown | 2,205 | 42 | 1.9 | 1.6 | 13 | 0.6 | 0.7 | .078 | .011 |
| **Exposure and risk** |  |  |  |  |  |  |  |  |  |
| Hospital admission | 16,946 | 395 | 2.3 | 2.3 | 326 | 1.9 | 2.1 | 0.115 | .000 |
| Surgical operations | 9,586 | 233 | 2.4 | 2.3 | 218 | 2.3 | 2.6 | 0.077 | .000 |
| Blood transfusion | 3,557 | 74 | 2.1 | 2.0 | 96 | 2.7 | 2.8 | 0.681 | .000 |
| Dental procedure | 23,220 | 500 | 2.2 | 2.1 | 299 | 1.3 | 1.4 | 0.767 | 0.089 |
| Skin piercing | 7,451 | 189 | 2.5 | 2.4 | 130 | 1.7 | 1.9 | 0.073 | .000 |
| Skin tattoo | 1,243 | 36 | 2.9 | 2.5 | 22 | 1.8 | 2.3 | 0.532 | 0.283 |
| IV drug use | 45 | 0 | 0.0 | 0.0 | 4 | 7.4 | 15.4 | .266) | .000 |
| Promiscuous sexual behavior | 193 | 3 | 1.6 | 1.3 | 3 | 1.6 | 2.6 | .464 | .814 |
| HBV Vaccination | 13,242 | 167 | 1.3 | 1.1 | 147 | 1.1 | 1.0 | .000 | 0.376 |
| HBV contact | 1,423 | 57 | 4.0 | 3.8 | 26 | 1.8 | 2.6 | .000 | .006 |
| History of jaundice | 2,421 | 88 | 3.6 | 3.6 | 51 | 2.1 | 2.5 | .000 | .000 |
